# Trastuzumab Produces Therapeutic Actions by Upregulating miR-26a and miR-30b in Breast Cancer Cells

**DOI:** 10.1371/journal.pone.0031422

**Published:** 2012-02-27

**Authors:** Takehiro Ichikawa, Fumiaki Sato, Kazuya Terasawa, Soken Tsuchiya, Masakazu Toi, Gozoh Tsujimoto, Kazuharu Shimizu

**Affiliations:** 1 Department of Nanobio Drug Discovery, Graduate School of Pharmaceutical Sciences, Kyoto University, Kyoto, Japan; 2 Department of Target Therapy Oncology, Graduate School of Medicine, Kyoto University, Kyoto, Japan; 3 Department of Pharmacogenomics, Graduate School of Pharmaceutical Sciences, Kyoto University, Kyoto, Japan; 4 Department of Breast Surgery, Graduate School of Medicine, Kyoto University, Kyoto, Japan; Baylor College of Medicine, United States of America

## Abstract

**Objective:**

Trastuzumab has been used for the treatment of HER2-positive breast cancer (BC). However, a subset of BC patients exhibited resistance to trastuzumab therapy. Thus, clarifying the molecular mechanism of trastuzumab treatment will be beneficial to improve the treatment of HER2-positive BC patients. In this study, we identified trastuzumab-responsive microRNAs that are involved in the therapeutic effects of trastuzumab.

**Methods and Results:**

RNA samples were obtained from HER2-positive (SKBR3 and BT474) and HER2-negetive (MCF7 and MDA-MB-231) cells with and without trastuzumab treatment for 6 days. Next, we conducted a microRNA profiling analysis using these samples to screen those microRNAs that were up- or down-regulated only in HER2-positive cells. This analysis identified miR-26a and miR-30b as trastuzumab-inducible microRNAs. Transfecting miR-26a and miR-30b induced cell growth suppression in the BC cells by 40% and 32%, respectively. A cell cycle analysis showed that these microRNAs induced G1 arrest in HER2-positive BC cells as trastuzumab did. An Annexin-V assay revealed that miR-26a but not miR-30b induced apoptosis in HER2-positive BC cells. Using the prediction algorithms for microRNA targets, we identified *cyclin E2* (*CCNE2*) as a target gene of miR-30b. A luciferase-based reporter assay demonstrated that miR-30b post-transcriptionally reduced 27% (p = 0.005) of the gene expression by interacting with two binding sites in the 3′-UTR of *CCNE2*.

**Conclusion:**

In BC cells, trastuzumab modulated the expression of a subset of microRNAs, including miR-26a and miR-30b. The upregulation of miR-30b by trastuzumab may play a biological role in trastuzumab-induced cell growth inhibition by targeting *CCNE2*.

## Introduction

The overexpression of HER2 has been reported in 20% to 30% of patients with breast cancer. The overall survival and time to relapse for patients whose tumors overexpressed HER2 were significantly shorter [1], [2]. The malignant phenotypes are also enhanced with HER2 overexpression. HER2-overexpressing tumors are also more likely to be resistant to treatment with tamoxifen and standard chemotherapy [3]–[5].

Trastuzumab (Herceptin) was designed to target the extra-cellular domain of HER2 and block its function, and is currently used in patients with HER2-positive breast and gastric cancers. The application of trastuzumab in the adjuvant and metastatic setting has been shown to prolong the survival of patients with HER2-positive breast cancer [6], [7]. The overall response rate was approximately 26–31% for trastuzumab monotherapy [8], [9], and 50–61% for trastuzumab-chemo combined regimens [6], [7]. Moreover, most patients with an initial response developed resistance to trastuzumab within one year [10]. Therefore, clarifying the molecular mechanisms of trastuzumab treatment will be beneficial to improve the treatment of HER2-positive breast cancer. For example, more fundamental knowledge about the mechanisms responsible for trastuzumab treatment would helpful in developing a monogram for tailoring trastuzumab treatment, and a novel agent for modulating the trastuzumab sensitivity of breast cancer cells.

According to accumulating reports, trastuzumab is thought to induce its therapeutic effects basically via two biological mechanisms: a direct effect by a blockade of the HER2 signal, and an induction of antibody-dependent cell-mediated cytotoxicity (ADCC). In terms of the direct therapeutic effects, trastuzumab binds to the extracellular domain of the HER2 molecule, and represses the signal transduction from the HER2 molecule by inhibiting the homo/hetero dimerization of HER2 and HER family members. Moreover, trastuzumab reduces the amount of HER2 on the breast cancer cell surface by promoting the internalization and cleavage of HER2 molecules. Therefore, trastuzumab blocks the downstream signal pathways from HER2 positive BC cells, including PI3K/Akt, MAPK, and mTOR pathways. However, little is known regarding the biological role of microRNAs in the trastuzumab therapeutic mechanism.

MiRNAs are a class of short, non-coding RNAs 18–25 nucleotides (nt) in length that are found in animal and plant cells. In 1993, the first miRNAs were recognized in *C. elegans*. In 2001, various small regulatory RNAs were discovered in plants and mammals, and were designated as “microRNA”s. As of today, 1921 human miRNAs are registered in the miRBASE database (Release 18, November, 2011). MiRNAs are involved in RNA interference (RNAi) machinery to regulate gene expression post-transcriptionally, and contribute to diverse physiological and pathophysiological functions, among them the regulation of developmental timing and pattern formation, the restriction of differentiation potential, cell signaling, and carcinogenesis.

In the present study, we screened for trastuzumab responsive microRNAs by utilizing microarray-based microRNA profiling. We identified miR-26a and miR-30b, which were induced in breast cancer cells by trastuzumab exposure, and played important biological roles in the trastuzumab therapeutic mechanism.

## Materials and Methods

### Cell lines and trastuzumab

Human mammary epithelial cells (HMEC, CC-2551, Lonza) were cultured using the medium supplied by the MEGM Bullet Kit (CC-3150, Lonza) at 37°C and 5% CO_2_. In this study, we used a total of 11 breast cancer cell lines. Among them, MCF7, MDAMB231,SKBR3, T47D (obtained from the American Type Culture Collection, ATCC), MDAMB453 (RCB1192, RIKEN BioResource Center), HMC-1-8, and MRK-nu-1 (JCRB0166 and JCRB0628 respectively, Health Science Research Resources Bank) were cultured in RPMI 1640 medium (Invitrogen) containing 10% FBS. BT474 (HTB-20, ATCC) Hs578T (86082104, European Collection of Cell Culture), YMB1E (TKG0440, Cell Resource Center for Biomedical Research, Tohoku University, identical to ZR-75-1,) were cultured in DMEM containing 10% FBS. Trastuzumab was kindly provided by Chugai Pharmaceutical Co., LTD. (Tokyo, Japan).

### DNA and RNA extraction from cells

The genomic DNA of breast cancer and HMEC cells was extracted using a DNeasy kit (Qiagen, Germany). Small RNA-preserved total RNA samples were extracted by a combination of Isogen reagent (Nippon Gene, Co., LTD. Japan) and a PureLink RNA mini kit (Invitrogen). The amount of DNA and RNA was measured by a Nanodrop spectrophotometer (ND-1), and the RNA quality of the samples was assessed by an Agilent's Bioanalyzer system (model-2100 and RNA 6000 nano kit).

### Assessment of HER2 amplification status in cell lines

The genome amplification status at the *HER2* locus in HMEC and the 11 breast cancer cell lines was assessed by quantitative genomic PCR. The amount of amplification at the *HER2* locus was normalized by the average amount of *NLK* and *ACACA* located between *HER2* and the centromere of chromosome 17. The genomic amount of the *HER2* locus relative to that in the HMEC cells represented the amount of *HER2* amplification in the cell lines. The sequence information used in this quantitative genomic PCR is listed in Table S1.

### Quantitative RT-PCR

The mRNA expression levels of genes such as *HER2* in the cell lines were assessed by SYBR green based quantitative RT-PCR (SYBR Green PCR Master Mix, Applied Biosystems, Carlsbad, CA). The RT-PCR data were normalized against the *GAPDH* expression in the cells. The sequence information used in this quantitative RT PCR was also listed in Table S1. The expression levels of individual miRNAs were determined by an ABI 7300 Sequence Detector™ (Applied Biosystems, Foster City, CA) with TaqMan MicroRNA Assay kits for hsa-miR-26a and 30b (Applied Biosystems). The miR-16 was used as an internal control to normalize the microRNA expression levels [11].

### MicroRNA expression profiling

To identify trastuzumab-inducible microRNAs, we performed microRNA expression profiling using microRNA microarray technology. The RNA samples were extracted from two *HER2*-positive cell lines (SKBR3 andBT474) and two *HER2*-negative cell lines (MCF7 and T47D), that were cultured with and without trastuzumab (4 µg/ml) for 144 hours. The global microRNA expression profiles of the 8 RNA samples were obtained using a Toray's microRNA microarray platform based on miRBase version 12 (3D-Gene miRNA oligo chip, Toray Industries Inc., Tokyo, Japan), as previously described [12]. Briefly, for each patient, 500 ng of total RNA derived from both tumor and non-tumor samples were labeled using a miRCURY LNA™ microRNA Power Labeling Kit Hy5 (Exiqon, Vedbaek, Denmark). The labeled samples were individually hybridized onto the DNA chip surface, and were incubated at 42°C for 16 hours. The washed and dried DNA chip in an ozone-free environment was scanned using a ProScanArray™ microarray scanner (PerkinElmer Inc. Waltham, MA). The obtained microarray images were then analyzed using Genepix Pro™ 4.0 software (Molecular Device, Sunnyvale, CA). In this study, the median values of the foreground signal minus the local background were calculated as the feature intensities.

### Transfection

The cells were plated at a density of 2×10^5^ cells per well in a 6-well format, or 5×10^3^ cells for a 96-well format 24 hours before the transfection. The microRNA precursor oligos, microRNA inhibitor, or negative control RNA (ncRNA) oligos (final concentration: 25 nM for mimic oligo, 40 nM for microRNA inhibitor) were transfected into cells using an X-tremeGENE siRNA Transfection Reagent (Roche). The medium was replaced eight hours after the transfection.

### WST-1 assay

We utilized a WST-1 assay for assessing the sensitivity of the cells to trastuzumab and the effect of the microRNA on cell proliferation. Regarding the sensitivity of the cells to trastuzumab, 5000 breast cancer cells were plated per 96-well plate on Day 0. From Day 1 to Day 6, the cells were exposed to trastuzumab at different concentrations of 0.0625, 0.125, 0.25, 0.5, 1, 2, 4, 8, 16, and 32 µg/ml, and the culture media containing trastuzumab were replaced every 72 hours. On Day 6, 10 µl of WST-1 reagent was added into each well. After 1 hour of incubation, the absorbance at 450 nm was measured by a microplate reader (Biorad, Hercules, CA). To assess the effect the microRNA on cell proliferation, a WST-1 assay was performed in the 96-well format at 72 hours after the microRNA/ncRNA transfection.

### Cell cycle assay

Flow cytometric analysis of the DNA content was performed to assess the effect of the microRNA on the cell cycle. Pn Day 0, miR-26a/30b precursor or ncRNA oligo (final concentration: 25 nM) was transfected into SKBR3 or BT474 cells in a 6-well format. On Day 3, the cells were fixed in 70% ethanol at −20°C. After washing with PBS, the cells were treated with RNase A and stained with propidium iodide (PI) using a Cellular DNA Flow Cytometric Analysis kit (Roche Diagnostics, Basel, Switzerland). The DNA content was evaluated using a FACS Calibur flow cytometer (BD Biosciences, San Jose, CA) with Modfit LT software (Verity Software House) for histogram analysis. Each experiment was performed in triplicate.

### Apoptosis assay

Annexin-V assays were performed for the detection of apoptotic cells. After the transfection of the microRNA precursors on Day 0, the cells were harvested on Day 3 and washed with PBS. The cells were then stained using an Annexin V-FITC Apoptosis Detection Kit I (BD Biosciences). The untreated cells served as a negative control for the double staining. The cells were analyzed immediately after staining using FACS Calibur flow cytometer and Cell Quest Pro software.

### Luciferase reporter assay for the association between the 3′UTR of target gene candidates and miR-30b

First, an EcoRI site was introduced into the XbaI site of the luciferase reporter vector pGL4.13 (Proamega, Madison, WI, USA) by ligation with the oligonucleotides 5′-CTAGACTGAATTC-3′ and 5′-CTAGGAATTCAGT-3′, yielding the pGL4.13EcoRI vector [13]. Second, the 3′-untranslated regions (UTRs) of the *CCNE2*, *cyclin A1* (*CCNA1*), and *cell division cycle 7* (*CDC7*) genes were amplified fromBT474 cells using the PCR primers listed in Table S2, and cloned into a pCR4-TOPO vector (Invitrogen). The cloned EcoRI fragments containing the putative miR-30b binding sites were then inserted into the EcoRI site of pGL4.13EcoRI, and were designated CCNE2-wt, CCNA1-wt, and CDC7-wt, respectively. Three derivative constructs o CCNE2-wt with mutations in the putative miR-30b-binding sites were generated using a Gene-Taylor Mutagenesis kit (Invitrogen) and the primers listed in Table S2, and were designated CCNE2-mut1, -mut2, and –mut1+2. All of the constructs were verified by direct sequencing. MicroRNA oligos or ncRNA were co-transfected with 200 ng each of the constructed reporter vector constructs and an internal control vector (pGL4.73, Promega) into HEK293 cells (5×10^4^ cells) in a 24-well format. Twenty-four hours later, the luciferase activity was measured using a dual-luciferase reporter assay system (Promega) and a Lumat LB9507 luminometer (Berthold Technologies, Germany). The firefly luciferase activities of the reporter constructs were normalized against the renilla luciferase activities of the internal control vector, The reduction ratio of the luciferase activity from the ncRNA-transfected samples was used as an index of the effect of the microRNAs on the post-transcriptional regulation of these 3 genes.

### Statistical analysis

The unpaired student-t test was used for evaluating whether a difference between two mean values was statistically significant. Matlab 2011a (Mathworks, MA, USA) or Microsoft Excel (Microsoft, Redmond, WA) software was used for these analyses, and a P-value of less than 0.05 was considered statistically significant.

## Results

### HER2 status and trastuzumab sensitivity in breast cancer cell lines

**Figure 1:**
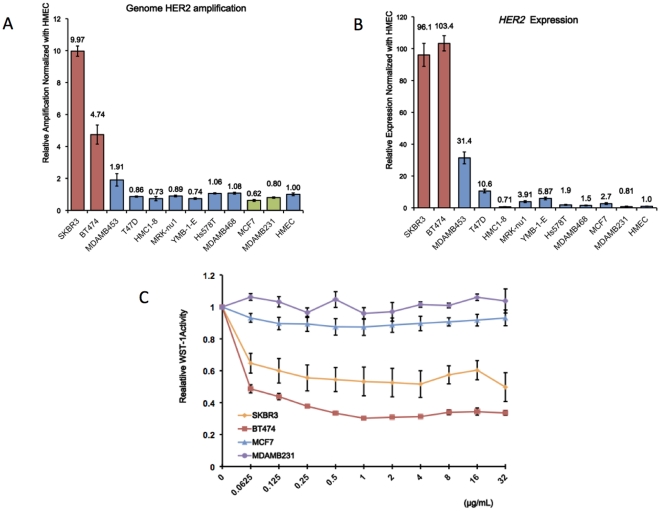
HER2-status of breast cancer cell lines. The genomic amplification (1A) and mRNA expression level (1B) of HER2 in 11 human breast cancer cell lines and normal human mammary epithelial cells (HMEC) were assessed using quantitative PCR and quantitative RT-PCR (n = 3). The mRNA abundance was normalized by the *GAPDH* expression levels. 1C: The trastuzumab sensitivity of SKBR3, BT474, MCF7, and MDA-MB-231 cells was determined using the WST-1 assay. The cells were incubated in trastuzumab-containing media at different concentrations for 144 hours, and then the absorbance at 450 nm was measured after a 2-hour incubation with WST-1 reagent. The ratio of the absorbance to that of the non-treated cells represented the trastuzumab sensitivity of cells.

To select HER2-positive and negative breast cancer cell lines, we determined the *HER2* status of the breast cancer cells in terms of genomic amplification and the mRNA expression of *HER2*. Figure 1A and 1B show the genome copy number in the *HER2* locus and the mRNA expression levels of *HER2* gene assessed by quantitative PCR, respectively. Among the 11 breast cancer cells, SKBR3 andBT474 exhibited marked genomic amplification and the overexpression of *HER2*, and MDA-MB-453 had a moderate level of *HER2* overexpression. Thus, for further study, we chose SKBR3 and BT474 as *HER2*-positive cells and MCF7 and MDA-MB-231 as *HER2*-negative cells.

The WST-1 assays showed that even very low concentrations of trastuzumab significantly reduced cell proliferation in SKBR3 andBT474 cells by 40–60%, whereas the proliferation of MCF7 and MDA-MB-231 cells was not affected by trastuzumab exposure (Fig. 1C). Thus, trastuzumab exposure directly reduced the growth of *HER2*-positive cells.

### Identification of trastuzumab-responsive microRNAs

In this study, we hypothesized that some of the trastuzumab-inducible/reducible microRNAs would play roles in the molecular mechanisms responsible for the therapeutic effect of trastuzumab. To identify these microRNAs, we performed microRNA expression profiling analysis. First, the two *HER2*-positive and two *HER2*-negative breast cancer cell lines were exposed to trastuzumab at a concentration of 4 µg/mL for six days. The control treatment consisted of PBS. Thus, a total of 8 RNA samples were extracted from these cells, and were subjected to microRNA profiling analysis. Second, the obtained microRNA profiling data were normalized by a quantile normalization method, and filtered using the criterion that the microRNA signals before or after trastuzumab exposure for each of the four cells, SKBR3, BT474, MCF7, and MDA-MB-231 cells, should be more than 6 in log2 transformed value. After this filtration, 94 microRNAs were subjected to further screening. All normalized and raw data from the microarray is available in Minimum Information about Microarray Gene Experiment (MIAME)-compliant format via the Gene Expression Omnibus (http://www.ncbi.nlm.nih.gov/geo). The accession numbers (GSM-numbers) are currently in the registration process. Third, the expression of trastuzumab-responsive microRNAs should not be changed by trastuzumab exposure in *HER2*-negative breast cancer cell lines MCF7 and MDA-MB-231. We eliminated those microRNAs, which had a more than 1.5-fold up/down-regulation in the MCF7 or MDA-MB-231 cells. Thus, 71 microRNAs remained. Fourth, the relative fold-change (fold change of microRNAs – average fold change of the microRNAs in MCF7 and MBA-MD-231 cells) of the remaining microRNAs, and microRNAs with more than a 1.5-fold up/down-regulation, were listed in Table 1.

**Table 1:** Trastuzumab responsive microRNAs.

Rank	microRNA	RFC* in SKBR3	microRNA	RFC* in BT474	microRNA	Mean RFC*
Up-regulated microRNAs						
1	miR-663	2.6847	miR-1246	3.5825	miR-1246	2.9505
2	miR-1228-5p	2.5757	miR-26a	2.3305	miR-26a	2.1640
3	miR-1246	2.4300	miR-125a-5p	1.7146	miR-1228-5p	2.0419
4	miR-21	2.2558	miR-23a	1.6897	miR-663	1.9317
5	miR-26b	2.0749	miR-30c	1.6455	miR-125a-5p	1.6925
6	miR-98	2.0446	miR-1228-5p	1.6187	miR-1908	1.6487
7	miR-26a	2.0093	miR-30b	1.6076	miR-23a	1.6303
8	miR-195	1.9996	miR-100	1.5625	miR-28-5p	1.6176
9	miR-28-5p	1.8877	miR-30d	1.5330	miR-26b	1.5799
10	miR-1908	1.8548			miR-100	1.5768
11	miR-29b	1.7555			miR-21	1.5335
12	miR-1268a	1.6898			miR-29b	1.5211
13	miR-125a-5p	1.6707				
14	miR-100	1.5913				
15	miR-23a	1.5730				
16	miR-149*	1.5439				
Down-regulated microRNAs						
1	none		miR-296-5p	0.4668	miR-296-5p	0.6417
2			miR-1308	0.4889		
3			miR-1280(d)	0.5604		
4			miR-18a	0.5609		
5			miR-425	0.5631		
6			miR-210	0.5670		
7			miR-720	0.5995		
8			miR-125a-3p	0.6064		
9			miR-494	0.6352		
10			miR-187*	0.6501		

MicroRNAs with more than 1.5-fold change in HER2-positive cells but not in HER2-negative cells.

* : RFC, relative fold change = (Fold change of miR) – (average fold change of the miR in MCF7 and MBA-MD-231).

**Figure 2:**
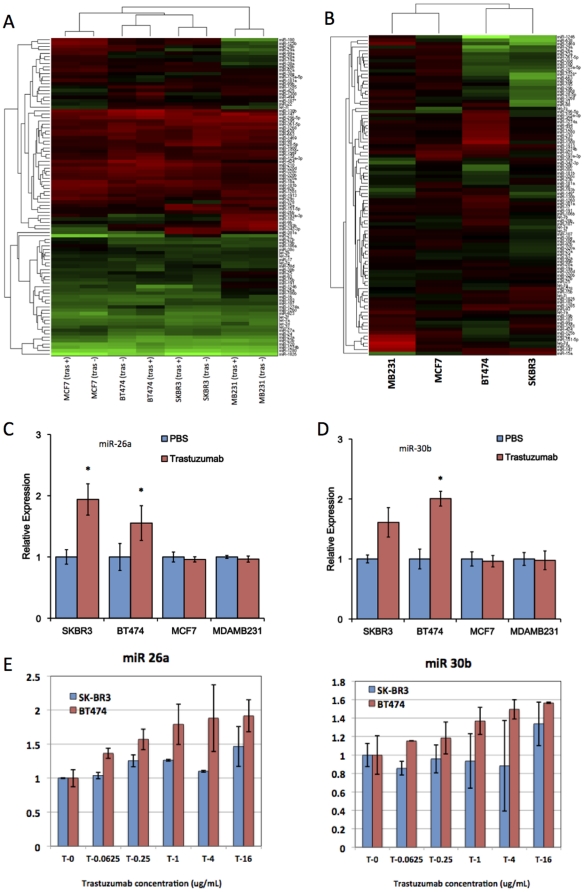
Identification of trastuzumab-responsive microRNAs. 2A: A heat map and clustergram of the expression profile of 71 pre-filtered microRNAs. The red and green represent higher and lower expression levels, respectively. (tras +): with trastuzumab treatment, (tras −): without trastuzumab treatment. 2B: A heatmap and clustergram of the fold-change of microRNA expression by trastuzumab treatment. The red and green represent up- and down-regulation. 2C and 2D: The expression levels of miR-26a (2C) and miR-30b (2D) were validated by qRT-PCR (n = 3). The data are shown as microRNA expression levels relative to a control treatment (PBS). 2E: The expression level of miR-26a and miR-30b in different trastuzumab concentrations was measured (n = 2). The microRNA expression levels were normalized against miR-16. All bars and error bars represent means ± SEM. *: p<0.05.

Trastuzumab exposure upregulated 16 and 9 microRNAs in SKBR3 and BT474 cells, whereas it down-regulated 0 and 10 microRNAs, respectively. As shown in a clustergram of 94 prefiltered microRNAs (Figure 2A), all pairs of the same cells with versus without trastuzumab treatment were clustered most closely, which indicated that the trastuzumab treatment changed microRNA profile slightly. The first branch of the clustergram divided *HER2*-positive and HER2-negative cells. This clustergram shows that the microRNA profile was reflected by the HER2-characteristics ofSKBR3, BT474, MCF7, and MDA-MB-231 cells, indicating that this profiling analysis worked well. The height of the last branch in the clustergram for the *HER2*-positive cells was higher than that of the *HER2*-negative cells, indicating that the *HER2*-positive cells had more altered microRNA expression than the *HER2*-negative cells. A heatmap and the clustergram in Figure 2B illustrated the fold-change pattern of the four cells following trastuzumab treatment.

Among the listed microRNAs, we selected seven microRNAs (miR-18a, miR-21, miR-26a, miR-26b, miR-30b, miR98 and miR-210) to validate the array-based expression data by Taqman quantitative RT-PCR (Figure 2C, 2D, S1). Most of the microRNAs showed consistent results with the array data. In particular, miR-26a and miR-30b in both cells were significantly upregulated in trastuzumab dose-dependent manner (Figure 2E). Interestingly, 3 out of 5 miR-30 family members (miR-30a∼e) were upregulated in the BT474 cells following trastuzumab exposure. Therefore, in this study, we focused on miR-26a and miR-30b for a further functional study.

A list of microRNAs which expression were altered only in HER2-negative cells is shown in Table S3. These microRNAs. The changes in these microRNAs could help to identify non-specific side effects of trastuzumab.

### Cell growth suppressive effects of miR26a and miR-30b

**Figure 3:**
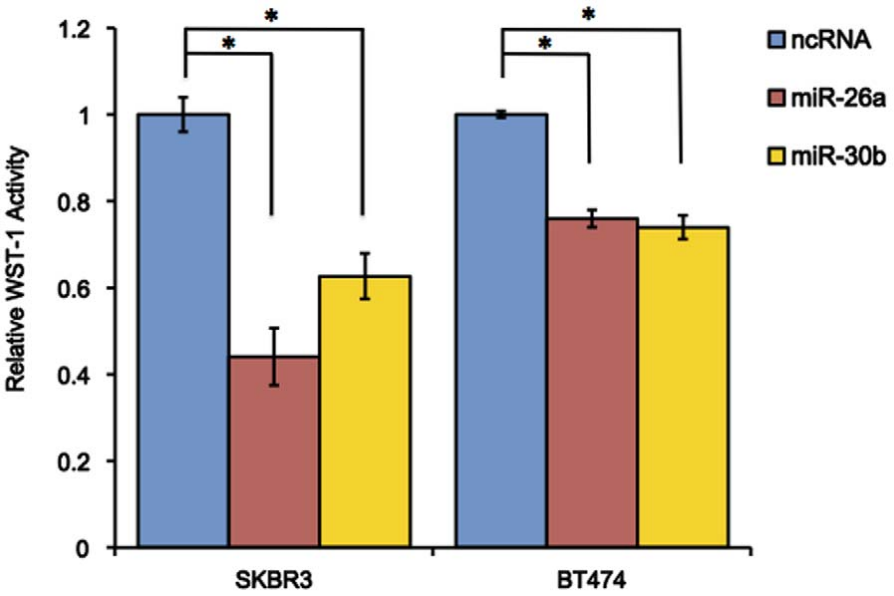
Effects of miR-26a and miR-30b on cell proliferation. The cells were transfected with negative control RNA (ncRNA), miR-26a, or miR-30b. At 72 hours after the transfection, the amount of viable cells was assessed by the WST-1 assay. The WST-1 activity values were normalized against that of the ncRNA-treatment. All bars and error bars represent means ± SEM (n = 4). *: p<0.05.

Using WST-1 assay, we examined whether miR-26a and miR-30b had growth suppressive effects. Six days after transfection, miR-26a significantly reduced the proliferation ofSKBR3 andBT474 cells by 56% and 24%, whereas miR-30b inhibited 37% and 26% of the cell growth, respectively (Figure 3, p<0.05).

**Figure 4:**
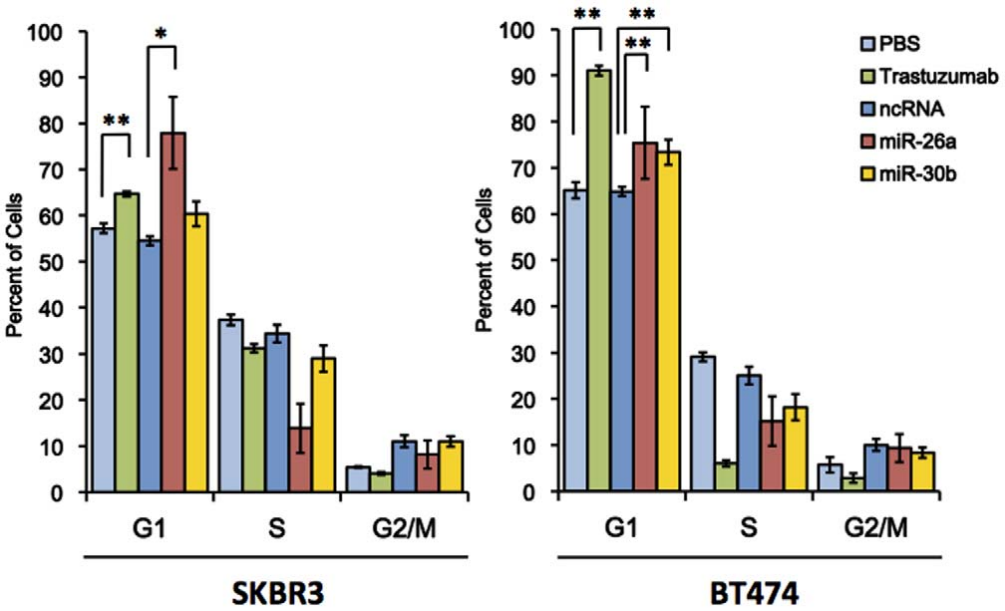
Effects of miR-26a and miR-30b on the cell cycle. The PI-stained DNA content of the cells was evaluated using a FACS Calibur (BD Biosciences) at 72 hours after transfection. All bars and error bars represent means ± SEM (n = 6). *: p<0.05, **: p<0.005.

Next, we checked whether the mechanisms responsible for this cell growth suppression by miR-26a and miR-30b included changes in the cell cycle and apoptosis. The proportion of cells in the G1 phase increased from 57% to 64% in theSKBR3 cells, and from 65% to 91% in the BT474 cells (Figure 4, p<0.005), and that in the S phase decreased from 37% to 31%, and from 29% to 6%, respectively. Thus, the trastuzumab treatment induced G1 arrest in both cell types. The transfection of miR-26a also showed a 22% (p = 0.14) and 11% (p = 0.0002) increment of the G1 proportion, and a 20% (p = 0.005) and 10% (p = 0.0005) decrement in the S phase in SKBR3 andBT474 cells, respectively. In contrast, the G2/M phase had no significant changes in both cell types. miR-30b also increased the G1 phase by 6% and 8%, and decreased the S phase by 5% and 7%, respectively, whereas the G2/M phase did not change. Thus, exogenous miR-26a and miR-30b induced G1 arrest in SKBR3 and BT474 cells.

**Figure 5:**
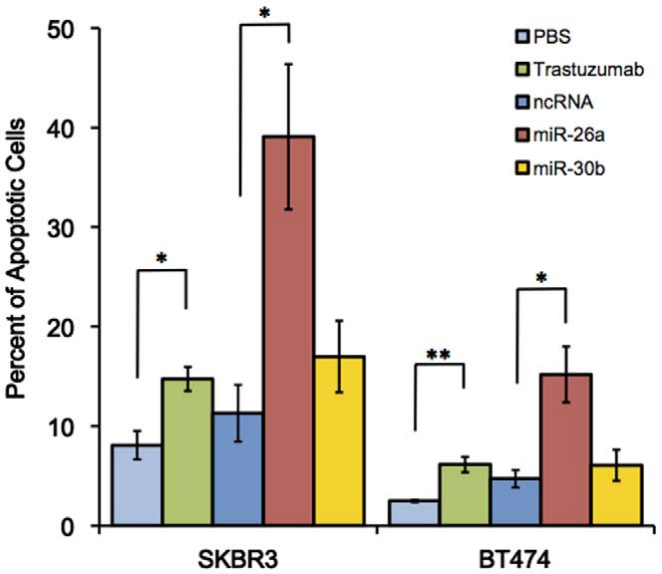
Effects of miR-26a and miR-30b on apoptosis. The apoptotic cells were detected using FITC-Annexin V at 72 hours after microRNA transfection. The percentage of Annexin V-FITC positive cells to the total cells was shown in the bar graphs. All bars and error bars represent means ± SEM (n = 4). *: p<0.05, **: p<0.005.

Using the Annexin-V assay, we also examined whether apoptosis was involved in the cell growth suppression induced by miR-26a and miR-30b. The trastuzumab treatment significantly increased the portion of apoptotic cells from 8.1% to 14.7% (Figure 5, p = 0.012), and from 2.5% to 6.1% (p = 0.003) inSKBR3 and BT474 cells, respectively. The transfection of miR-26a induced apoptosis in both cell types, as compared with the non-targeting control microRNA (from 11.3% to 39%, p = 0.012 in SKBR3, and from 4.7% to 15.2%, p = 0.012 in BT474 cells), whereas miR-30b did not show any significant effect on apoptosis.

### Identification of target genes against miR-30b

**Figure 6:**
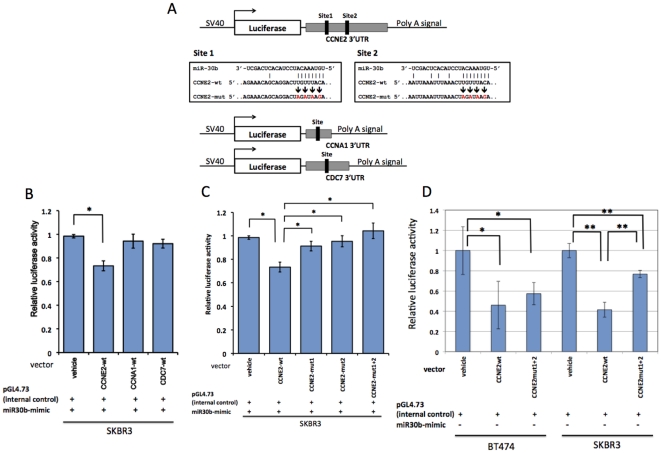
*CCNE2* is a direct target of miR-30b in breast cancer cells. 6A: A diagram of the 3′UTR-containing reporter constructs for *CCNE2*, *CCNA1*, and *CDC7* and their derivatives. The 3′UTRs of the three genes were inserted just downstream of the firefly luciferase gene in the pGL4.13 vector (wt). Next, the mutated derivatives (mut1, mut2, and mut1+2) of CCNE2-wt were generated by inserting mutations into two putative binding sites corresponding to the seed-sequence of miR-30b. 6B and 6C: SKBR3 and BT474 cells were co-transfected with reporter constructs, internal control vector (pGL4.73), and synthetic miR-30b oligomer. 6D: assessment of endogenous microRNA's inhibitory effects to *CCNE2*. Only reporter constructs and pGL4.73 were transfected into SKBR3 and BT474 cells. Twenty-four hours after the transfection, the reporter luciferase activity was measured. The data were shown as the luciferase activity relative to that of vehicle (pGL4.13+pGL4.73) transfection. All bars and error bars represent means ± SEM (n = 3). *: p<0.05, **: p<0.005.

In the present study, we tried to identify the target mRNAs of miR-30b that were related to miR-30b-induced G1 arrest. First, we utilized three different algorithms for predicting the microRNA targets, TargetScan5.1 (http://www.targetscan.org/), miRanda (http://www.microrna.org), and PicTar (http://www.pictar.org/). Among the putative target genes listed by all of three prediction engines, we selected 3 cell cycle-related genes, *CCNE2*, *CCNA1*, and *CDC7*. We then examined whether these three genes were actually regulated by miR-30b or not, using luciferase reporter vectors containing the 3′UTR of these genes (Figure 6A). *CCNE2* and *CDC7* have two and one putative binding sites for miR-30b in the conserved regions of the 3′-UTR, respectively, whereas *CCNA1* possesses one miR-30b binding site in a poorly conserved region of the 3′-UTR.

Among the three reporter constructs with the wild-type 3′-UTR of these genes, miR-30b reduced the luciferase activity only of the CCNE2-wt construct (27% reduction, p = 0.005, Figure 6B). To confirm whether miR-30b was associated with the predicted binding sites, we generated three derivative constructs with mutations at the miR-30b binding sites (Figure 6A). These mutations abolished the post-transcriptional repressive effect of miR-30b (Figure 6C), which indicated that miR-30b interacts directly with both binding sites. However, transfecting excessive exogenous microRNA may lead an artificial effect. Thus, we tried to assess suppressive effect of microRNAs at the endogenous level. First, we used microRNA inhibitor for co-transfection (Figure S2), which did not show any significant effect. We speculated that other miR-30 family members with the same seed sequence could compensate the function of blocked miR-30b. Alternatively, we transfected reporter constructs without miR-30b mimic oligos into cells (Figure 6D). Endogenous microRNAs suppressed 54–59% of reporter actively by binding *CCNE2*. When mutated construct at both two miR-30b binding sites was used, 11–35% reporter actively was recovered, which represented the total suppressive effect of endogenous miR-30 family through *CCNE2* 3′UTR.

Figure S3 showed that exogenous miR-30b mimic-oligos and inhibitors did not change mRNA levels of *CCNE2*. One of possible reasons is that miR-30b may regulate *CCNE2* only by translational inhibition. Another reason would be the change of cell cycle proportion of treated cells. The *CCNE2* is upregulated in G1 phase of cell cycle in a normal condition. Because introduction of miR26a/30b oligos increase G1 phase, *CCNE2* expression will be affected both by change of cell cycle phase proportion and post-transcriptional suppression due to these microRNAs. Because the two luciferase genes in reporter vector and internal control vector (pGL4.73) were driven by the same promoter (SV40), this system can assess the post-transcriptional regulation without any cell cycle-related bias.

## Discussion

Recent evidence has shown that altered patterns of miRNA expression are correlated with carcinogenesis, malignant potential, prognosis [14], and the treatment response of various human cancers. In breast cancers, a high expression level of miR-10b [15] and miR-21 [16] are associated with metastasis and a poor outcome. Regarding the treatment response of breast cancer, the in vitro experiments showed that miR-34a [17] and miR-221/222 [18], [19] are involved in the actions of docetaxel and tamoxifen, and that multidrug resistance–associated protein (MRP) was targeted by miR-7, mir-326, and miR-345 [20], [21]. However, little has been reported in terms of microRNAs associated with the molecular mechanisms of trastuzumab treatment. This was the aim of this study.

At the beginning of this study, we confirmed the genome amplification and mRNA expression status of HER2 among the 11 breast cancer cell lines. SKBR3 andBT474 cells have high levels of genomic amplification and mRNA expression, and also exhibited trastuzumab sensitivity. This finding was also consistent with previous studies [22], [23].

To screen the microRNAs related to the mechanisms of trastuzumab treatment, we initially set two selection criteria. The first one was microRNAs that were differentially expressed between trastuzumab sensitive and resistant *HER2*-positive breast cancer cells, and the second was microRNAs that were induced or reduced by trastuzumab treatment only in *HER2*-positive cells. For the former criterion, all of the *HER2*-positive breast cancer cells were trastuzumab sensitive. Furthermore, to establish trastuzumab-resistant *HER2*-positive cells, we administered trastuzumab to SKBR3 andBT474 cells at a concentration of 32 µg/mL for more than three months. However, these long-treated cells gained only 10–20% resistance as compared to the original cells, which were still moderately sensitive, similar to the MDA-MB-453 cells (data not shown). This was the reason why we chose the latter criteria in this study.

Using microarray-based microRNA profiling analysis and these screening criteria, we obtained a list of trastuzumab responsive microRNAs, as shown in Table 1. The validation of the RT-PCR demonstrated that most of the seven microRNAs had expression results consistent with the microarray data. Among the seven microRNAs, we focused on miR-26a as a microRNA up-regulated in bothSKBR3 andBT474 cells, and on miR-30b, because three out of five miR-30 family members were up-regulated inBT474 cells.

A down-regulation of miR-26a has been observed in various human malignancies, such as thyroid [24], liver cancer [25] and rhabdomyosarcoma [26], indicating that miR-26a is a tumor-suppressor microRNA. This study showed that the up-regulation of miR-26a by trastuzumab induced G1 arrest and apoptosis, which was consistent with previous observations. Some papers have reported the genes that were targeted by miR-26a, and are related to cell cycle and apoptosis. miR-26a regulated the cell cycle by targeting *cyclin D2* and *CCNE2* [27], and induced apoptosis by silencing the *enhancer of zeste*, *drosophila*, *homolog 2 (EZH2)*, and *metadherin* (*MTDH*) [28].

The expression of miR-30b was suppressed in invasive bladder cancer [29] and lung squamous cell carcinoma [30], as compared with superficial bladder cancer and the adjacent normal lung tissues, respectively. This suggests that miR-30b is also a tumor-suppressor microRNA. Transfecting with miR-30b had a cell growth suppressive effect and induced G1 cell cycle arrest, which was in agreement with the previous reports. Although information regarding the target genes of miR-26a was available, little has been known in terms of miR-30 target. Therefore, we screened the target genes of miR-30b that contributed to the miR-30b-induced G1 arrest. In this study, we demonstrated that miR-30b interacts directly with two binding sites in the 3′-UTR of *CCNE2*, and suppresses the expression of CCNE2. Cyclin E as well as Cyclins A and D are required for mammalian cells to transverse G1 and enter the S phase. Cyclin E1 and E2 activate cyclin-dependent kinase 2 (CDK2) by forming a CCNE-CDK2 complex [31], and initiate DNA synthesis. Therefore, it was a reasonable finding that the downregulation of CCNE2 by miR-30b induced G1 arrest. In Table 1, miR-30c and miR-30d were up-regulated by trastuzumab in BT474 cells. These miR-30 family members share the same sequence, 5′-GUAAACA-3′, in their seed regions. Thus, CCNE2 would be reduced in trastuzumab-treated BT474 cells not only by an up-regulation of miR-30b and miR-26a, but also by that of miR-30c/d. Recently, Scaltriti et al. demonstrated that gene amplification and overexpression of CCNE1 were associated with resistance of trastuzumab treatment for breast cancer [32], suggesting that cell cycle check-point system by CCNE is a key function for HER2-positive breast cancer. Thus, our finding that trastuzumab-inducible miR-26a/30b are regulating *CCNE2* was consistent with the their finding.

As shown in table 1, miR-125a-5p level was up-regulated both in SKBR3 and BT474 cells by trastuzumab exposure. Nishida et al. recently showed that miR-125a-5p targets HER2, and that it acts synergistically with trastuzumab in gastric cancer [33]. Our result suggested that the same mechanism would underlie trastuzumab therapy for breast cancer.

However, generally, each microRNA can target potentially hundreds of genes. Therefore, the cell cycle/apoptosis may not be the only processes affected/regulated by miR-26a/miR-30b. In addition, this study is not suggesting that the suppressive effect in endogenous level of these microRNA is a main mechanism of trastuzumab therapeutic effect. Direct blocking effect of HER2 signal pathway is still the major mechanism of trastuzumab therapy, and alteration of microRNA expression could play a supporting role in the downstream of HER2 signal.

On the other hand, it is largely unknown how miR-26a and miR-30b are up-regulated by trastuzumab treatment. One possible explanation of this phenomenon is regulation via c-myc (MYC) [34]. MYC is located downstream of the HER2 signal pathway [35]. Thus, trastuzumab treatment can reduce the levels of phospho-MYC [36]. According to the MYC ChIP-seq data registered in the UCSC genome browser [37], there are c-myc binding peaks around the transcriptional start sites of the miR-26a primary genes (*CTDSPL* in chromosome 3p22.2 and *CTDSP2* in 12q14.1). Actually, a report showed that miR-26a was repressed by MYC [38]. Furthermore, there is a MYC-binding site in a CpG island located upstream of the intergenic and polycistronic miR-30b and miR-30d. Thus, we hypothesized that inactivation of *MYC* may upregulate miR-30b/d expression. However, knock down of *MYC* by siRNA down regulated miR-30b expression (Figure S4 and S5). Therefore, unknown mechanisms rather than *MYC* upregulate miR-30b expression in trastuzumab treatment.

The present study demonstrated that a subset of microRNAs played a biological role in the mechanisms responsible for trastuzumab's antitumor effects. This finding suggests that trastuzumab-resistant *HER2*-positive breast cancer cells could be sensitized to trastuzumab therapy by modulating the expression of these microRNAs [39]. Alternatively, some microRNAs would be biomarkers to predict the treatment response of trastuzumab.

In summary, trastuzumab treatment for breast cancer cells modulated the expression of a subset of microRNAs, including miR-26a and miR-30b. The up-regulation of miR-30b by trastuzumab may play a biological role in trastuzumab-induced cell growth inhibition by targeting *CCNE2*.

## Supporting Information

Figure S1 **Taqman RT-PCR to validate the microarray results.** The fold change in the log2 values are shown in the Y-axis.
(TIFF)

Figure S2 **Effect of microRNA inhibitors on the CCNE2-3′UTR reporter assay.** SKBR3 cells were transfected with CCNE2-wt construct and microRNA inhibitors to assess the suppressive effect of endogenous microRNAs. Twenty-four hours after the transfection, the reporter luciferase activity was measured. NTC: non-specific control oligos. The data were shown as the luciferase activity relative to that of NC. All bars and error bars represent means ± SEM (n = 3). *: p<0.05, **: p<0.005.
(TIFF)

Figure S3 **Effect of knockdown and overexpression of miR-26a and 30b on CCNE2 mRNA expression.** SKBR3 and BT474 cells were transfected with microRNA mimic oligos and inhibitors. Twenty-four hours after the transfection, mRNA level of *CCNE2* was measured by quantitative RT-PCR. *GAPDH* mRNA level was used for normalization of data. The data using inhibitor and mimic oligo were shown as relative expression to each non-specific control (NC) oligo. All bars and error bars represent means ± SEM (n = 4).
(TIFF)

Figure S4 **Knocking down efficiency of** ***MYC*** **by siRNA.** *MYC* mRNA level was measured by quantitative RT-PCR after 72 hours later than control siRNAs (siCont) or 4 different siRNAs (Qiagen) against *MYC* gene that were purchased from Qiagen, designated as siMYC1, siMYC5, siMYC7, and siMYC8. The siMYC5 and siMYC7 were selected for further study. Y-axis: *MYC* expression level relative to siCont transfection. All bars and error bars represent means ± SEM (n = 3).
(TIFF)

Figure S5 **Effect of** ***MYC*** **knockdown on miR26a and miR30b expression.** The microRNA (miR26a and 30b) expression level was measured by Taqman RT-PCR system after 72 hours later than the transfection of siCont or siMYCs. Y-axis: microRNA expression level relative to that of siCont transfection. All bars and error bars represent means ± SEM (n = 3).
(TIFF)

Table S1 **Primer sequences for quantitative PCR.**
(DOCX)

Table S2 **Primer sequences for generating luciferase reporter constructs.**
(DOCX)

Table S3 **Trastuzumab responsive microRNAs in HER2-negative cells.** MicroRNAs with more than 1.5-fold change in HER2-negative cells but not in HER2-positive cells. *: RFC, relative fold change = (Fold change of miR) – (average fold change of the miR in SKBR3 and BT474)
(DOCX)

## References

[1] Slamon DJ, Godolphin W, Jones LA, Holt JA, Wong SG (1989). Studies of the HER-2/neu proto-oncogene in human breast and ovarian cancer.. Science.

[2] Hynes NE, Stern DF (1994). The biology of erbB-2/neu/HER-2 and its role in cancer.. Biochim Biophys Acta.

[3] Rosen PP, Lesser ML, Arroyo CD, Cranor M, Borgen P (1995). Immunohistochemical detection of HER2/neu in patients with axillary lymph node negative breast carcinoma. A study of epidemiologic risk factors, histologic features, and prognosis.. Cancer.

[4] Carlomagno C, Perrone F, Gallo C, De Laurentiis M, Lauria R (1996). c-erb B2 overexpression decreases the benefit of adjuvant tamoxifen in early-stage breast cancer without axillary lymph node metastases.. J Clin Oncol.

[5] Gusterson BA, Gelber RD, Goldhirsch A, Price KN, Save-Soderborgh J (1992). Prognostic importance of c-erbB-2 expression in breast cancer. International (Ludwig) Breast Cancer Study Group.. J Clin Oncol.

[6] Slamon DJ, Leyland-Jones B, Shak S, Fuchs H, Paton V (2001). Use of chemotherapy plus a monoclonal antibody against HER2 for metastatic breast cancer that overexpresses HER2.. N Engl J Med.

[7] Marty M, Cognetti F, Maraninchi D, Snyder R, Mauriac L (2005). Randomized phase II trial of the efficacy and safety of trastuzumab combined with docetaxel in patients with human epidermal growth factor receptor 2-positive metastatic breast cancer administered as first-line treatment: the M77001 study group.. J Clin Oncol.

[8] Nishimura R, Okumura Y, Arima N (2008). Trastuzumab monotherapy versus combination therapy for treating recurrent breast cancer: time to progression and survival.. Breast Cancer.

[9] Vogel CL, Cobleigh MA, Tripathy D, Gutheil JC, Harris LN (2002). Efficacy and safety of trastuzumab as a single agent in first-line treatment of HER2-overexpressing metastatic breast cancer.. J Clin Oncol.

[10] Esteva FJ, Valero V, Booser D, Guerra LT, Murray JL (2002). Phase II study of weekly docetaxel and trastuzumab for patients with HER-2-overexpressing metastatic breast cancer.. J Clin Oncol.

[11] Davoren PA, McNeill RE, Lowery AJ, Kerin MJ, Miller N (2008). Identification of suitable endogenous control genes for microRNA gene expression analysis in human breast cancer.. BMC Mol Biol.

[12] Sato F, Tsuchiya S, Terasawa K, Tsujimoto G (2009). Intra-platform repeatability and inter-platform comparability of microRNA microarray technology.. PLoS One.

[13] Terasawa K, Ichimura A, Sato F, Shimizu K, Tsujimoto G (2009). Sustained activation of ERK1/2 by NGF induces microRNA-221 and 222 in PC12 cells.. FEBS J.

[14] Sato F, Hatano E, Kitamura K, Myomoto A, Fujiwara T (2011). MicroRNA profile predicts recurrence after resection in patients with hepatocellular carcinoma within the Milan Criteria.. PLoS One.

[15] Ito T, Tanaka E, Kadowaki T, Kan T, Higashiyama M (2007). An ultrasensitive new DNA microarray chip provides gene expression profiles for preoperative esophageal cancer biopsies without RNA amplification.. Oncology.

[16] Qian B, Katsaros D, Lu L, Preti M, Durando A (2009). High miR-21 expression in breast cancer associated with poor disease-free survival in early stage disease and high TGF-beta1.. Breast Cancer Res Treat.

[17] Kastl L, Brown I, Schofield AC (2011). miRNA-34a is associated with docetaxel resistance in human breast cancer cells.. Breast Cancer Res Treat.

[18] Miller TE, Ghoshal K, Ramaswamy B, Roy S, Datta J (2008). MicroRNA-221/222 confers tamoxifen resistance in breast cancer by targeting p27Kip1.. J Biol Chem.

[19] Zhao JJ, Lin J, Yang H, Kong W, He L (2008). MicroRNA-221/222 negatively regulates estrogen receptor alpha and is associated with tamoxifen resistance in breast cancer.. J Biol Chem.

[20] Liang Z, Wu H, Xia J, Li Y, Zhang Y (2010). Involvement of miR-326 in chemotherapy resistance of breast cancer through modulating expression of multidrug resistance-associated protein 1.. Biochem Pharmacol.

[21] Pogribny IP, Filkowski JN, Tryndyak VP, Golubov A, Shpyleva SI (2010). Alterations of microRNAs and their targets are associated with acquired resistance of MCF-7 breast cancer cells to cisplatin.. Int J Cancer.

[22] Marches R, Uhr JW (2004). Enhancement of the p27Kip1-mediated antiproliferative effect of trastuzumab (Herceptin) on HER2-overexpressing tumor cells.. Int J Cancer.

[23] Dubska L, Andera L, Sheard MA (2005). HER2 signaling downregulation by trastuzumab and suppression of the PI3K/Akt pathway: an unexpected effect on TRAIL-induced apoptosis.. FEBS Lett.

[24] Visone R, Pallante P, Vecchione A, Cirombella R, Ferracin M (2007). Specific microRNAs are downregulated in human thyroid anaplastic carcinomas.. Oncogene.

[25] Ji J, Shi J, Budhu A, Yu Z, Forgues M (2009). MicroRNA expression, survival, and response to interferon in liver cancer.. N Engl J Med.

[26] Ciarapica R, Russo G, Verginelli F, Raimondi L, Donfrancesco A (2009). Deregulated expression of miR-26a and Ezh2 in rhabdomyosarcoma.. Cell Cycle.

[27] Kota J, Chivukula RR, O'Donnell KA, Wentzel EA, Montgomery CL (2009). Therapeutic microRNA delivery suppresses tumorigenesis in a murine liver cancer model.. Cell.

[28] Zhang B, Liu XX, He JR, Zhou CX, Guo M (2011). Pathologically decreased miR-26a antagonizes apoptosis and facilitates carcinogenesis by targeting MTDH and EZH2 in breast cancer.. Carcinogenesis.

[29] Wszolek MF, Rieger-Christ KM, Kenney PA, Gould JJ, Silva Neto B (2011). A MicroRNA expression profile defining the invasive bladder tumor phenotype.. Urol Oncol.

[30] Gao W, Shen H, Liu L, Xu J, Xu J (2011). MiR-21 overexpression in human primary squamous cell lung carcinoma is associated with poor patient prognosis.. J Cancer Res Clin Oncol.

[31] Gudas JM, Payton M, Thukral S, Chen E, Bass M (1999). Cyclin E2, a novel G1 cyclin that binds Cdk2 and is aberrantly expressed in human cancers.. Mol Cell Biol.

[32] Scaltriti M, Eichhorn PJ, Cortes J, Prudkin L, Aura C (2011). Cyclin E amplification/overexpression is a mechanism of trastuzumab resistance in HER2+ breast cancer patients.. Proc Natl Acad Sci U S A.

[33] Nishida N, Mimori K, Fabbri M, Yokobori T, Sudo T (2011). MicroRNA-125a-5p is an independent prognostic factor in gastric cancer and inhibits the proliferation of human gastric cancer cells in combination with trastuzumab.. Clin Cancer Res.

[34] Boominathan L (2010). The guardians of the genome (p53, TA-p73, and TA-p63) are regulators of tumor suppressor miRNAs network.. Cancer Metastasis Rev.

[35] Kanehisa M (2011).

[36] Le XF, Pruefer F, Bast RC (2005). HER2-targeting antibodies modulate the cyclin-dependent kinase inhibitor p27Kip1 via multiple signaling pathways.. Cell Cycle.

[37] Rozowsky J, Euskirchen G, Auerbach RK, Zhang ZD, Gibson T (2009). PeakSeq enables systematic scoring of ChIP-seq experiments relative to controls.. Nat Biotechnol.

[38] Sander S, Bullinger L, Klapproth K, Fiedler K, Kestler HA (2008). MYC stimulates EZH2 expression by repression of its negative regulator miR-26a.. Blood.

[39] Blower PE, Chung JH, Verducci JS, Lin S, Park JK (2008). MicroRNAs modulate the chemosensitivity of tumor cells.. Mol Cancer Ther.

