# Supplementary figures and images for: Trastuzumab Produces Therapeutic Actions by Upregulating miR-26a and miR-30b in Breast Cancer Cells
Source: PLoS One. 2012 Feb 27;7(2):e31422. doi: 10.1371/journal.pone.0031422 (PMC3288043; doi:10.1371/journal.pone.0031422)

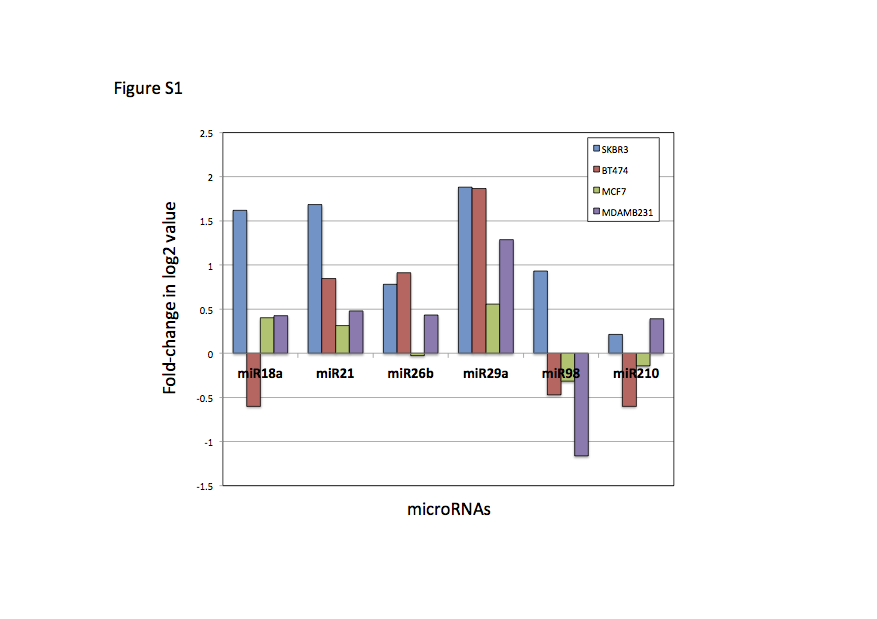

Supplement: Figure S1 — Taqman RT-PCR to validate the microarray results. The fold change in the log2 values are shown in the Y-axis. (TIFF) [file pone.0031422.s001.tiff]

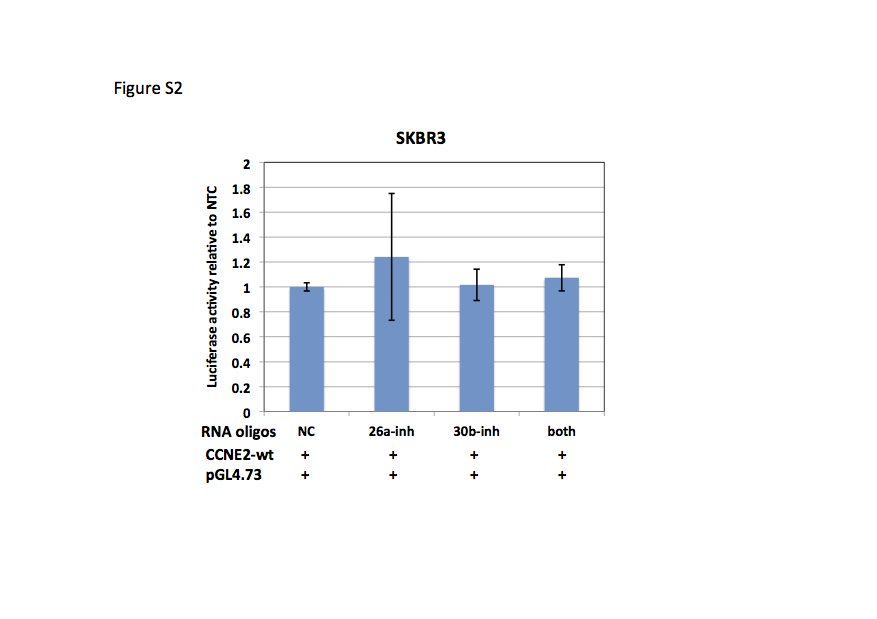

Supplement: Figure S2 — Effect of microRNA inhibitors on the CCNE2-3′UTR reporter assay. SKBR3 cells were transfected with CCNE2-wt construct and microRNA inhibitors to assess the suppressive effect of endogenous microRNAs. Twenty-four hours after the transfection, the reporter luciferase activity was measured. NTC: non-specific control oligos. The data were shown as the luciferase activity relative to that of NC. All bars and error bars represent means ± SEM (n = 3). *: p<0.05, **: p<0.005. (TIFF) [file pone.0031422.s002.tiff]

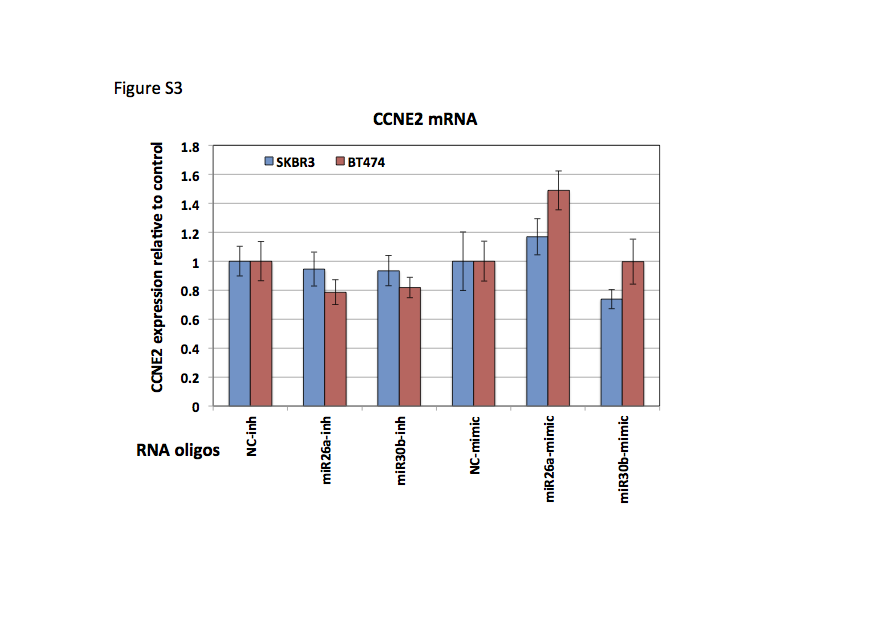

Supplement: Figure S3 — Effect of knockdown and overexpression of miR-26a and 30b on CCNE2 mRNA expression. SKBR3 and BT474 cells were transfected with microRNA mimic oligos and inhibitors. Twenty-four hours after the transfection, mRNA level of CCNE2 was measured by quantitative RT-PCR. GAPDH mRNA level was used for normalization of data. The data using inhibitor and mimic oligo were shown as relative expression to each non-specific control (NC) oligo. All bars and error bars represent means ± SEM (n = 4). (TIFF) [file pone.0031422.s003.tiff]

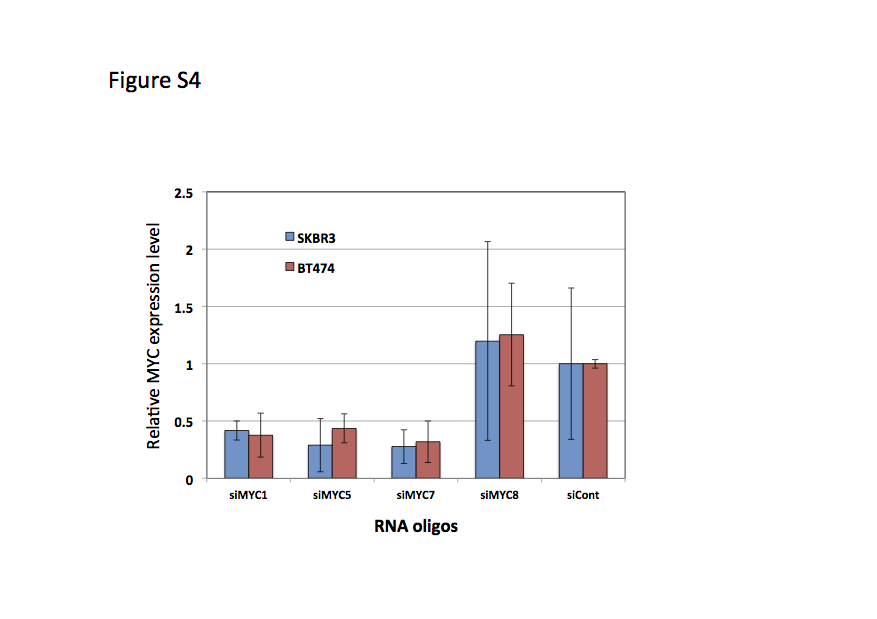

Supplement: Figure S4 — Knocking down efficiency of MYC by siRNA. MYC mRNA level was measured by quantitative RT-PCR after 72 hours later than control siRNAs (siCont) or 4 different siRNAs (Qiagen) against MYC gene that were purchased from Qiagen, designated as siMYC1, siMYC5, siMYC7, and siMYC8. The siMYC5 and siMYC7 were selected for further study. Y-axis: MYC expression level relative to siCont transfection. All bars and error bars represent means ± SEM (n = 3). (TIFF) [file pone.0031422.s004.tiff]

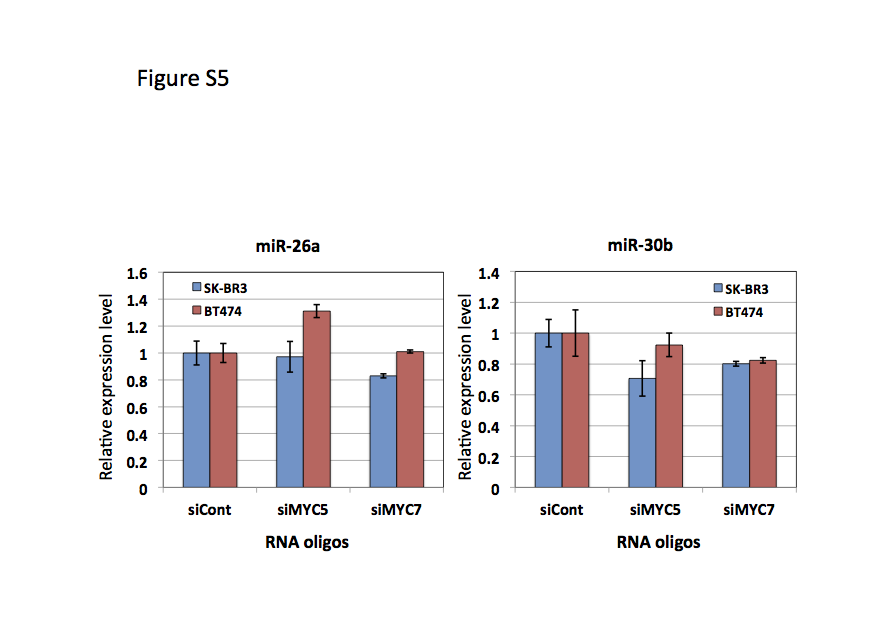

Supplement: Figure S5 — Effect of MYC knockdown on miR26a and miR30b expression. The microRNA (miR26a and 30b) expression level was measured by Taqman RT-PCR system after 72 hours later than the transfection of siCont or siMYCs. Y-axis: microRNA expression level relative to that of siCont transfection. All bars and error bars represent means ± SEM (n = 3). (TIFF) [file pone.0031422.s005.tiff]
