# Supplementary material for: Trastuzumab Produces Therapeutic Actions by Upregulating miR-26a and miR-30b in Breast Cancer Cells
Source: PLoS One. 2012 Feb 27;7(2):e31422. doi: 10.1371/journal.pone.0031422 (PMC3288043; doi:10.1371/journal.pone.0031422)
Supplement: Table S1 — Primer sequences for quantitative PCR. (DOCX) [file pone.0031422.s006.docx]

Table S1. Primer sequences for quantitative PCR.

| PCR type | Primer name | Sequence (5’ -> 3’) |
| --- | --- | --- |
| Genomic | g-HER2-F | CTGAACTGGTGTATGCAGATTGC |
| Genomic | g-HER2-R | TTCCGAGCGGCCAAGTC |
| Genomic | g-NLK-F | ACACGCTCACCCCAAAATTA |
| Genomic | g-NLK-R | GCAGATGTACCGCCATTGTA |
| Genomic | g-ACACA-F | TTGCTGCAACTAAAGGAGAGC |
| Genomic | g-ACACA-R | ACTCCTGATGGCCTCAAACT |
| RT | rt-HER2-exon2-3-F | GGGAAACCTGGAACTCACCT |
| RT | rt-HER2-exon2-3-R | GACCTGCCTCACTTGGTTGT |
| RT | rt-CCNE2-exon7-8-F | GCTTCAACTCATTGGAATTACCT |
| RT | rt-CCNE2-exon7-8-R | TCTTCACTGCAAGCACCATC |
| RT | rt-MYC-exon2-3-F | CTGGTGCTCCATGAGGAGA |
| RT | rt-MYC-exon2-3-R | CTCTGACCTTTTGCCAGGAG |
| RT | rt-GAPDH-F | GAAGGTGAAGGTCGGAGTC |
| RT | rt-GAPDH-R | GGAAGATGGTGATGGGATTTC |
