# Supplementary material for: Trastuzumab Produces Therapeutic Actions by Upregulating miR-26a and miR-30b in Breast Cancer Cells
Source: PLoS One. 2012 Feb 27;7(2):e31422. doi: 10.1371/journal.pone.0031422 (PMC3288043; doi:10.1371/journal.pone.0031422)
Supplement: Table S2 — Primer sequences for generating luciferase reporter constructs. (DOCX) [file pone.0031422.s007.docx]

Table S2. Primer sequences for generating luciferase reporter constructs.

| Primer name | Sequence (5’ -> 3’) |
| --- | --- |
| CCNE2-3’UTR-F | AGAAGATAACTAAGCAAACAAGT |
| CCNE2-3’UTR-R | TCCTTATCTCACAATGGAGGAA |
| CCNA1-3’UTR-F | GTTTCTGAATGGAAGCACTT |
| CCNA1-3’UTR-R | TCTAACACATGGTCATAAGACAAGC |
| CDC7-3’UTR-F | TAATGGATCTTCATTTAATGTTTACT |
| CDC7-3’UTR-R | AGATCTGGATGGCTCTACGC |
| CCNE2-site1mut-F | ACAGCAGGACTAGATAAGAAAGATGTCTTC |
| CCNE2-site1mut-R | GAAGACATCTTTCTTATCTAGTCCTGCTGT |
| CCNE2-site2mut-F | AAATTTAAACTAGATAAGAAAGGTTTAGTTTTG |
| CCNE2-site2mut-R | CAAAACTAAACCTTTCTTATCTAGTTTAAAT |
