# Supplementary material for: Trastuzumab Produces Therapeutic Actions by Upregulating miR-26a and miR-30b in Breast Cancer Cells
Source: PLoS One. 2012 Feb 27;7(2):e31422. doi: 10.1371/journal.pone.0031422 (PMC3288043; doi:10.1371/journal.pone.0031422)
Supplement: Table S3 — Trastuzumab responsive microRNAs in HER2-negative cells. MicroRNAs with more than 1.5-fold change in HER2-negative cells but not in HER2-positive cells. *: RFC, relative fold change = (Fold change of miR) – (average fold change of the miR in SKBR3 and BT474) (DOCX) [file pone.0031422.s008.docx]

Table S3. Trastuzumab responsive microRNAs in HER2-negative cells

| Rank | microRNA | RFC* in MCF7 | microRNA | RFC* in MDA-MB-231 | microRNA | Mean  RFC* |
| --- | --- | --- | --- | --- | --- | --- |
| Up-regulated microRNAs | | | | | | |
| 1 | miR-423-3p | 2.2680 | miR-148a | 1.6319 | miR-423-3p | 1.8765 |
| 2 | let-7c | 1.5734 | miR-342-3p | 1.5930 |  |  |
| 3 |  |  | miR-423-3p | 1.5526 |  |  |
| Down-regulated microRNAs | | | | | | |
| 1 | miR-193a-3p | 0.5685 | let-7d | 0.3317 | miR-16 | 0.5399 |
| 2 | miR-30a | 0.6591 | miR-16 | 0.3706 | let-7d | 0.5440 |
| 3 |  |  | miR-34a | 0.3932 | miR-22 | 0.5748 |
| 4 |  |  | miR-151-5p | 0.4059 | miR-151-5p | 0.5812 |
| 5 |  |  | miR-22 | 0.4373 | miR-34a | 0.5821 |
| 6 |  |  | miR-125b | 0.6188 |  |  |
| 7 |  |  | let-7a | 0.6499 |  |  |

MicroRNAs with more than 1.5-fold change in HER2-negative cells but not in HER2-positive cells

*: RFC, relative fold change = (Fold change of miR) – (average fold change of the miR in SKBR3 and BT474)
